# A Circular Network of Coregulated L-Threonine and L-Tryptophan Metabolism Dictates Acute Lower Limb Ischemic Injury

**DOI:** 10.7150/ijms.102177

**Published:** 2024-09-16

**Authors:** Liheng Li, Chengjiang Xiao, Hao Liu, Siliang Chen, Yinhong Tang, Hao Zhou, Guihua Jiang, Junzhang Tian

**Affiliations:** 1 Department of Interventional Radiology, The Affiliated Guangdong Second Provincial General Hospital of Jinan University, Guangzhou 510317, China.; 2 The Affiliated Guangdong Second Provincial General Hospital of Jinan University, Guangzhou 510317, China.; 3 Xianning Medical College, Hubei University of Science & Technology, Xianning 437000, China.; 4 Department of Radiography, The Affiliated Guangdong Second Provincial General Hospital of Jinan University, Guangzhou 510317, China.

**Keywords:** Lower limb ischemia, SLC6A19, L-threonine, and L-tryptophan.

## Abstract

Lower limb ischemia is characterized by reduced arterial perfusion in the lower limbs, leading to tissue ischemia and cell death. It is primarily caused by thrombosis and the rupture of arterial plaques, resulting in damage to ischemic muscle tissues. Metabolic processes are crucial in its development. Herein we combined single-cell data with metabolomics data to explore the pathways and mechanisms influencing lower limb ischemia. We analyzed single-cell and metabolomics data. In single-cell analysis, we identified different cell subpopulations and key regulatory genes, and biological enrichment analysis was performed to understand their functions and relationships. For metabolomics, mass spectrometry and chromatography techniques were employed to analyze metabolites in clinical samples. We performed differential analysis, correlation analysis, and Mendelian randomization to determine the relationships between key metabolites and genes. Nebl, Dapl1, Igfbp4, Lef1, Klrd1, Ciita, Il17f, Cd8b1, Il17a, Cd180, Il17re, Trim7, and Slc6a19 were identified to play a crucial role in lower limb ischemia. Important metabolites included L-threonine and L-tryptophan. The metabolism of L-threonine and L-tryptophan is linked to lower limb ischemia and thrombosis. B0AT1, encoded by *SLC6A19*, is closely related to these metabolites and appears to play a key role in lower limb ischemia development. Our analysis revealed the roles of key genes and metabolites in lower limb ischemia. These findings enhance our understanding of the pathogenesis of lower limb ischemia and provide new insights into its prevention and treatment.

## Introduction

Lower limb ischemia refers to the reduction in arterial perfusion in the lower limbs, leading to tissue ischemia and cell death. It requires prompt intervention or surgery to restore blood flow to the lower limbs 1. The incidence rate of acute lower limb ischemia in adults in the United States is approximately 0.2% 2. Despite early intervention, the amputation rate remains as high as 15% 3, 4. Therefore, lower limb ischemia has become an increasingly significant health threat.

The primary causes of lower limb ischemia are thrombosis and arterial plaque rupture 5. Thrombosis can result from vascular injury during surgery, increased risk of blood coagulation, and hemodynamic changes. Surgical instrument manipulation can cause mechanical damage to the vessel wall; further, stent implantation may stimulate and damage the vascular endothelium and vessel wall, activating the blood coagulation system and leading to thrombus formation 6, 7. In individuals with predisposing factors, hypertension can damage the vascular intima and cause inflammation, increasing the risk of blood coagulation 8, 9. High levels of cholesterol and triglycerides also elevate this risk 10, 11, while inherited coagulation disorders, such as factor V Leiden mutation and prothrombin gene mutation, make blood more prone to clotting, increasing the risk of thrombus formation 12, 13.

Hemodynamic changes are closely related to atherosclerosis and plaque rupture. Plaques form due to lipid deposition and macrophage enrichment within blood vessels 14, 15. As plaques develop, the blood flow passage gradually narrows, altering hemodynamics. When a plaque ruptures, collagen is exposed on the vascular wall, triggering platelet aggregation at specific receptors for collagen and coagulation factors. This accelerates hemodynamic changes and promotes thrombus formation 16, 17.

Lower limb ischemia also damages ischemic muscle tissue. Ischemia leads to a lack of oxygen and nutrient supply to muscle cells, affecting energy synthesis and metabolic processes 18. It also causes hypoxia in muscle tissue, disrupting energy production within cells, resulting in a lack of ATP and an increase in intracellular calcium ion concentration. This damages cell membrane integrity, causes mitochondrial dysfunction, and impairs intracellular transport 19.

Metabolic processes play a critical role in the development of lower limb ischemia. Increased expression of 6-phosphofructo-2-kinase/fructose-2, 6-bisphosphatase-3 in critical limb ischemia has been reported to enhance glycolytic metabolism, maintain mitochondrial function, alleviate pathological damage, and promote muscle tissue repair and recovery 20. In addition, metabolites affect thrombosis. Phenylacetylglutamine reportedly increases platelet function, enhances responses to various stimuli, and releases intracellular calcium ions, thus increasing platelet participation in thrombus formation 21, 22. The metabolism of precursor nutrients, such as choline and carnitine, by gut microbiota produces trimethylamine N-oxide. High levels of trimethylamine N-oxide can alter the function of platelets, induce the synthesis of coagulation factors in endothelial cells, and increase the incidence of vascular inflammation 23, 24, ultimately leading to limb thrombosis and lower limb ischemia.

Herein to further explore the impact of gene transcription and metabolites on lower limb ischemia, we analyzed single-cell data combined with metabolomics data. We aimed to investigate the specific pathways and mechanisms influencing the occurrence and development of lower limb ischemia.

## Methods and Materials

### Ethical statement

This study was performed in strict accordance with the recommendations in the Guide for the Care and Use of Laboratory Animals at Guangdong Second Provincial General Hospital. Animal procedures were performed according to protocols approved by the Animal Ethics Committee at Guangdong Second Provincial General Hospital (approval no.: 2024-KY-KZ-244-01).

### Single-cell data download, calculation, and clustering

We obtained the single-cell RNA sequencing dataset GSE150383 for lower limb ischemia from Meng S, Lv J, Chen K, and Cooke JP. This dataset includes single-cell sequencing expression profiles of one mouse sample at Day 0 and another one at Day 28. GSE150383 was downloaded from the Gene Expression Omnibus (GEO) database (https://www.ncbi.nlm.nih.gov/geo/) 25, 26. Single-cell RNA sequencing data were processed primarily using the Seurat package (v4.0) in R software. Quality control was performed to evaluate cell and gene expression quality. The NormalizeData function in Seurat was used for gene expression normalization, and the ScaleData function was used for gene expression scaling. Highly variable genes were identified using the FindVariableFeatures function. Dimensionality reduction, cell clustering, and visualization were performed using the RunPCA function 25, 27. The FindNeighbors and FindClusters functions were used to calculate the relationship between cells, and cells were clustered into different subgroups. The DimPlot function in Seurat was used to visualize cell clustering results and generate UMAP plots, and the heatmap package was used to create cell clustering heatmaps. Cell subgroup reannotation was performed to further explore the underlying mechanisms.

### Key cell subpopulation and regulatory gene detection

The FindAllMarkers function in Seurat was used to identify marker genes for each cell subgroup in GSE150383. The expression of these marker genes in each cell subgroup was compared using CellMarker 2.0 (http://bio-bigdata.hrbmu.edu.cn/CellMarker/) to determine cell types and improve cell type annotation 26, 27. To more accurately annotate cell subgroups, an extensive literature review was conducted to clarify the function and expression of marker genes in cells. Significant differentially expressed genes were identified for each cell subgroup and reannotated subgroups, which were considered key regulatory genes 15, 28.

### Biological function enrichment analysis

We used the clusterProfiler package to perform gene ontology (GO) analysis on the identified differentially expressed genes, annotating them in different functional categories (biological processes, molecular functions, and cellular components) to identify significantly enriched functional categories and understand their roles in biological processes 29. Moreover, the clusterProfiler package was used to perform Kyoto Encyclopedia of Genes and Genomes (KEGG) analysis on the identified differentially expressed genes and metabolites, analyzing their enrichment in KEGG pathways to identify genes and metabolites enriched in specific biological pathways and their functions in biological pathways 26, 30.

Metabolite set enrichment analysis was conducted on differentially expressed metabolites to explore their biological significance 28, 31. Four metabolite sets based on the KEGG database, including 84 human metabolism pathways, specific biological fluids, and disease-related pathways, were used to enrich the metabolome data and identify significantly different metabolite sets 32.

We also performed gene set enrichment analysis (GSEA) on highly variable genes and genes within transcriptional trajectories identified in each cell subpopulation. We used the clusterProfiler package in R software to rank these genes based on their expression patterns and differential expression 31. The predefined gene set library from the Molecular Signatures Database was then used to calculate the enrichment of these genes in the ranked gene list. Finally, the enrichment score and statistical significance were calculated to evaluate the reliability of our results 33, 34.

### Protein-protein interaction (PPI) network analysis

The STRING database (https://string-db.org/) was used to search for PPI of proteins involved in metabolic pathways 13. Only experimentally validated and database-analyzed confirmed PPIs with a confidence cutoff threshold of 0.7 were considered. Cytoscape v3.8.2 was used for PPI network construction, analysis, and visualization 34, 35.

### Metabolomics analysis

To further elucidate the role of metabolites, untargeted metabolomics analysis was performed on seven individuals: four patients with lower limb ischemia and three healthy controls. Quality control of metabolites was performed using the coefficient of variation (CV) and hierarchical clustering. The CV, calculated as the ratio of the standard deviation to the mean, reflects the degree of data dispersion 35. The empirical cumulative distribution function was used to analyze the frequency of CV values for below reference thresholds, indicating data stability. Hierarchical clustering analysis was performed on different comparison group samples to generate a cluster tree showing sample similarity 7, 36.

Orthogonal partial least squares discriminant analysis (OPLS-DA) model validation for metabolites was conducted. Evaluation parameters included R2X, R2Y, and Q2, where R2X and R2Y represent the goodness of fit for the X and Y matrices, respectively, and Q2 represents the predictability of the model. Higher values close to 1 indicate a more stable and reliable model, with Q2 > 0.5 considered effective and Q2 > 0.9 indicating an excellent model. Differential metabolite analysis was performed. A volcano plot of differential metabolites was generated, along with scatter plots of differential metabolites showing relative content differences and radar plots of the top 10 metabolites with the largest absolute log_2_FC 24, 37.

Pearson correlation analysis was conducted to calculate the correlation between metabolites 36. The covariance of variables was calculated to determine the strength and direction of their linear relationship. A correlation test determined the significance of the correlation coefficient, and a significance threshold of P < 0.05 was used to visualize the top 50 metabolites. MetaboAnalyst (https://www.metaboanalyst.ca/) was used to explore the relationship between differential metabolites and genes 9, 38.

### Validation data acquisition and analysis

GSE124595 provided by Wang X *et al.* and GSE152139 were downloaded from the GEO database (https://www.ncbi.nlm.nih.gov/geo/). The probes of these two datasets were processed to remove invalid probes and where multiple probes were used for the same gene, the median value was used as the gene expression level to generate expression profiles. The expression differences of SLC6A19 in these two datasets were then validated 38, 39.

### Mendelian randomization (MR) analysis

MR analysis was performed to investigate the relationship between differential metabolites and lower limb ischemia. To avoid linkage imbalance, a standard of kb = 10,000 and r2 = 0.001 was used when aggregating single nucleotide polymorphisms (SNPs). In addition, p < 5 × 10^-8^ was set as the genome-wide significance threshold, and palindromic SNPs were removed. The MR analysis mainly employed the classical inverse-variance weighted (IVW) method, which utilizes the effect sizes and inverse variances of each genetic variant to weight the effects, reducing estimation bias caused by heterogeneity and combining the estimated effect sizes of multiple variants 39.

Four statistical methods were simultaneously used: the weighted median estimator model, weighted model-based method 40, MR-Egger regression model, and simple model. The weighted median estimator model calculates the median effect sizes of multiple genetic variant sites and combines them using weighted averaging to estimate the comprehensive effect size 4. The MR-Egger regression model evaluates bias and symmetry of causal effect estimation using Egger regression concepts. The simple model extracts basic genetic information and association rules, providing insights into genotype frequency distribution and phenotype correlation. Harmonization was also performed to address incompatible allele genes SNPs and palindromic SNPs. Heterogeneity testing and MR-Egger regression testing were conducted using the IVW method, with p values for the correlation between L-threonine and lower limb ischemia (0.99) and L-tryptophan and lower limb ischemia (0.99) indicating no heterogeneity. Horizontal pleiotropy in the MR analysis was checked using the intercept value in MR-Egger, with p > 0.05 suggesting negligible multidirectionality. Finally, leave-one-out analyses were performed to assess the consistency of our results.

## Results

### Single-cell data analysis

**Figure 1:**
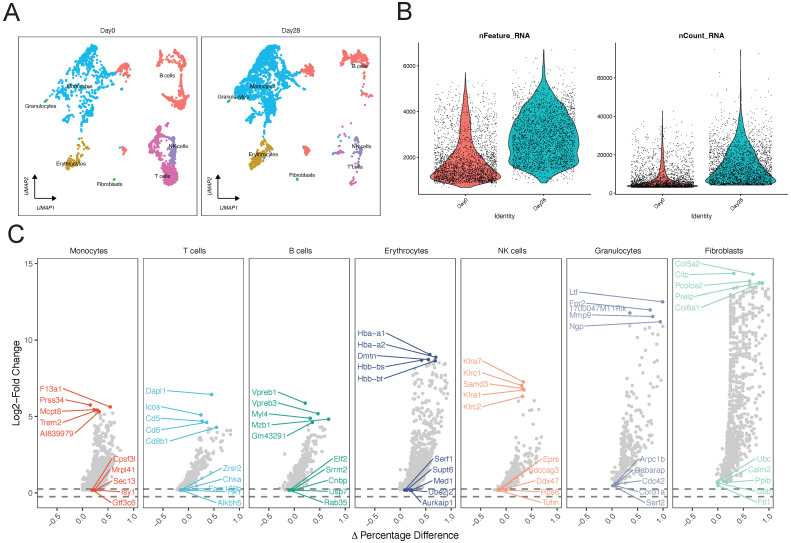
** Single-cell analysis of GSE150383 to identify core subgroups and key regulatory genes.** (A) Cell clustering at Day 0 and Day 28. (B) Quality control of GSE150383. (C) Key regulatory genes of the core subgroups in GSE150383.

A significant improvement in data structure was observed after data filtering. We then identified the core subgroups of GSE150383 at Day 0 and Day 28, which represent critical time points. GSE150383 could be divided into seven cell subgroups both at Day 0 and Day 28: monocytes, T cells, B cells, erythrocytes, natural killer (NK) cells, granulocytes, and fibroblasts (Figure 1A-B). The expression profiles of these cell subgroups differed between the time points, indicating changes in cell subgroups with the progression of lower limb ischemia. We also identified key regulatory genes for each of these seven subgroups. For monocytes, these included F13a1, Prss34, and Mcpt8; for T cells, Dapl1, Icos, and Cd5; for B cells, Vpreb1, Vpreb3, and Myl4; for erythrocytes, Hba-a1, Hba-a2, and Dmtn; for NK cells, Klra7, Klrc1, and Samd3; for granulocytes, Ltf, 1700047M11Rik, and Fpr2; and for fibroblasts, Col5a2, Cilp, and Pcolce2 (Figure 1C).

**Figure 2:**
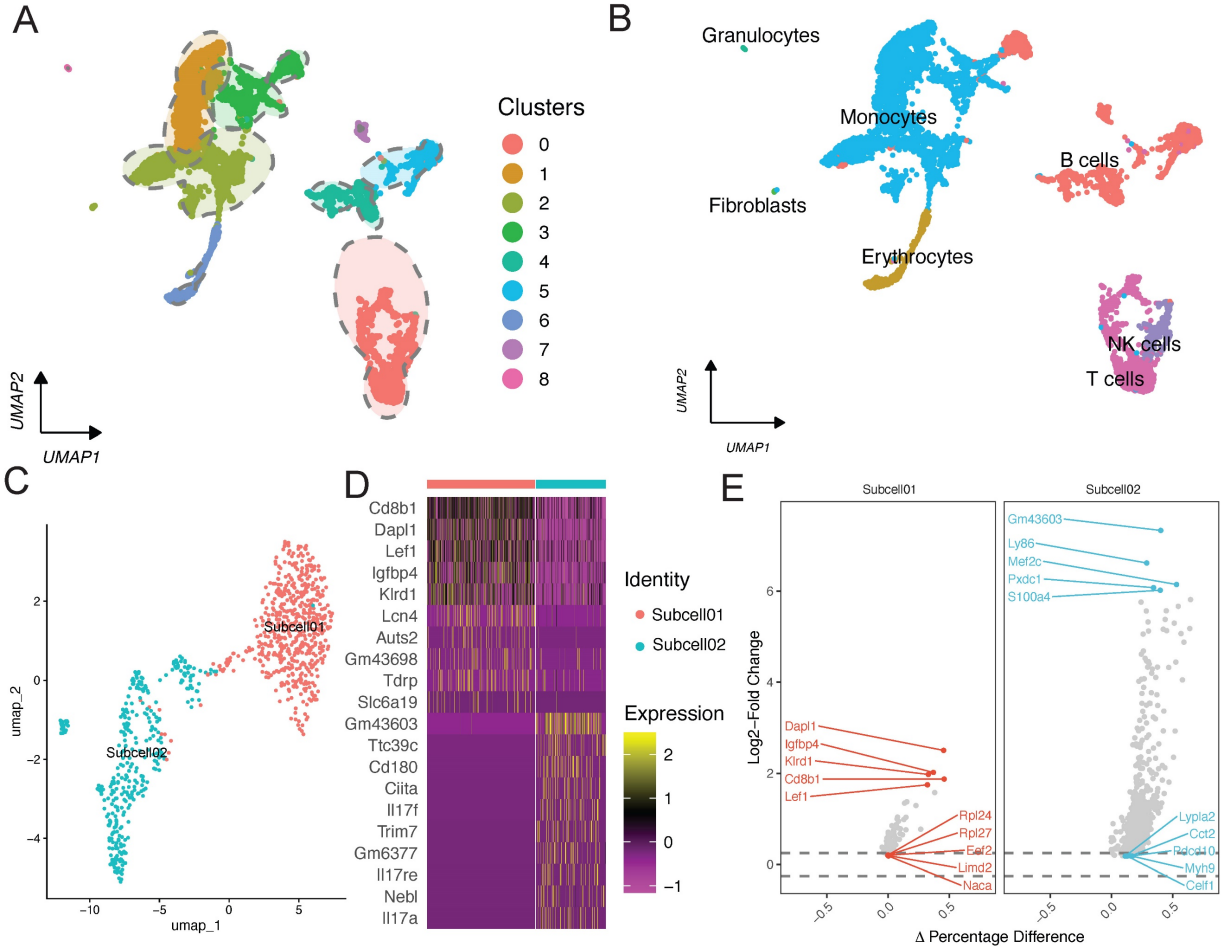
** Single-cell analysis of GSE150383, reannotation of core subpopulations, and identification of key regulatory genes.** (A-B) Identification of core subpopulations in GSE150383, regardless of time. (C) Reannotation of the T cell subpopulation. (D) Gene heatmap of the reannotated subpopulations within the T cell subpopulation. (E) Identification of key regulatory genes within the reannotated subpopulations.

Initially, we identified the key subpopulations in GSE150383 at Day 0 and Day 28 (Figure 1B; Figure 2A). However, considering the overall nature of the data, we also identified core subpopulations in GSE150383 without considering time. We found that GSE150383 could be divided into the same seven cell subgroups as previously identified (Figure 2B). Subsequently, we observed the expression changes at Day 0 and Day 28 and found that T cells exhibited the most significant changes. Therefore, we focused on reannotating the T cell subpopulation (Figure 2C). Our analysis revealed that the T cell subpopulation could be further divided into two subgroups. Heatmaps were generated for differentially expressed genes in these two subgroups, and key regulatory genes were identified within each subgroup (Figure 2D). The key regulatory genes in the first subgroup included Dapl1, Igfbp4, and Klrd1, while those in the second subgroup included Gm43603, Ly86, and Mef2c (Figure 2E).

### Transcriptional and functional features of key subpopulations reveal heterogeneity in lower limb ischemic injury

**Figure 3:**
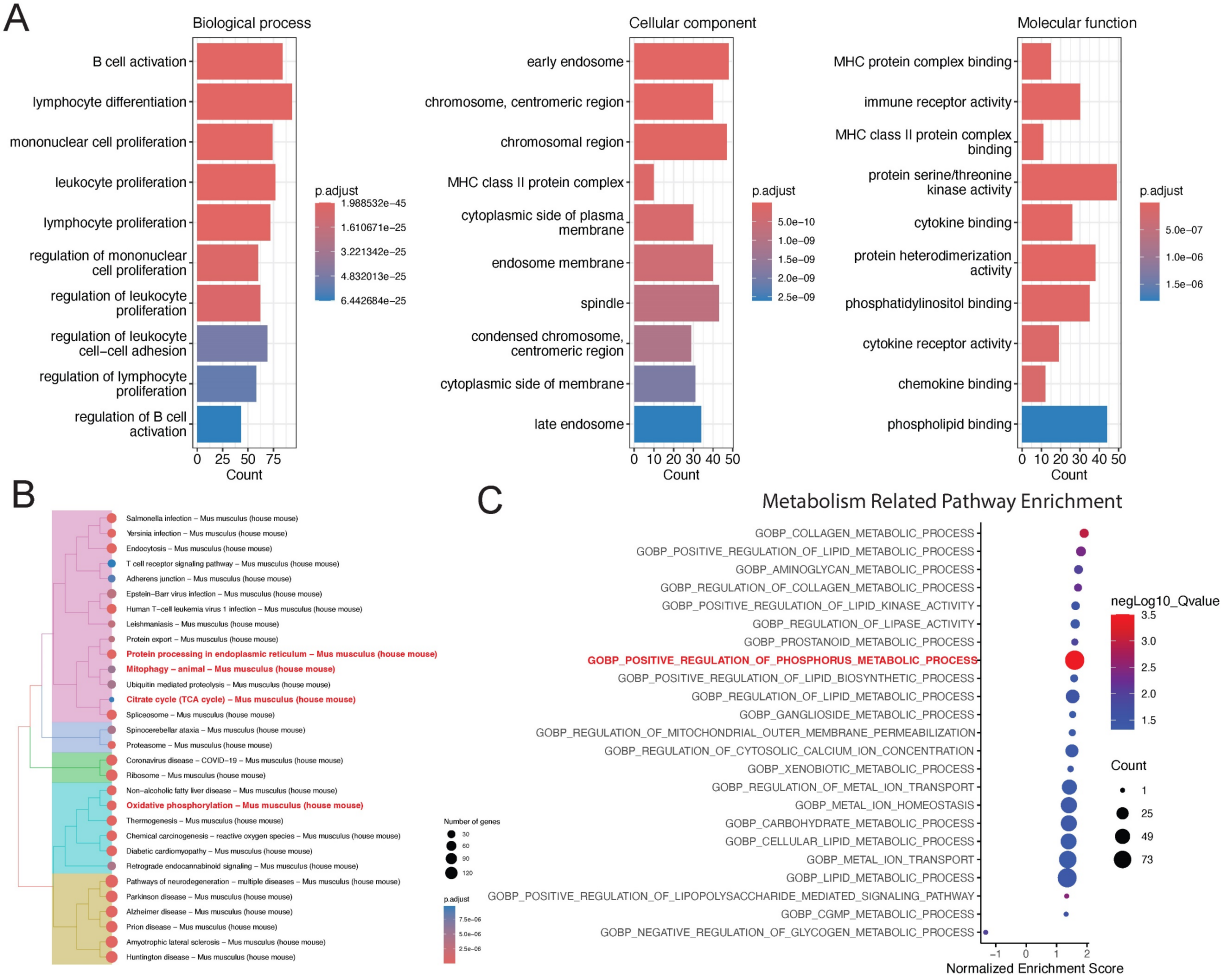
** Functional enrichment analysis of the T cell subpopulation.** (A) GO and (B) KEGG pathway enrichment analyses. (C) GSEA of metabolism-related pathways in the T cell subpopulation.

To understand the biological enrichment pathways of the T cell subpopulation, we performed GO analysis on differentially expressed genes identified in this subpopulation (Figure 3A). Biological process enrichment analysis showed their involvement in processes such as B cell activation and lymphocyte differentiation; cellular component enrichment analysis showed their involvement in processes such as “early endosome” and “chromosome, centromeric region”; and molecular function enrichment analysis showed their involvement in processes such as MHC protein complex binding and immune receptor activity.

We also performed KEGG pathway enrichment analysis on differentially expressed genes in the T cell subpopulation, which revealed that the most significant pathways were protein processing in endoplasmic reticulum, citrate cycle (TCA cycle), and oxidative phosphorylation (Figure 3B). Finally, we performed GSEA on differentially expressed genes in the T cell subpopulation to realize that the most significant pathway was “GOBP: positive regulation of phosphorus metabolic process.” Accordingly, we believe that metabolic changes play a key role in the development of lower limb ischemia (Figure 3C).

### Functional enrichment analysis based on GSEA

**Figure 4:**
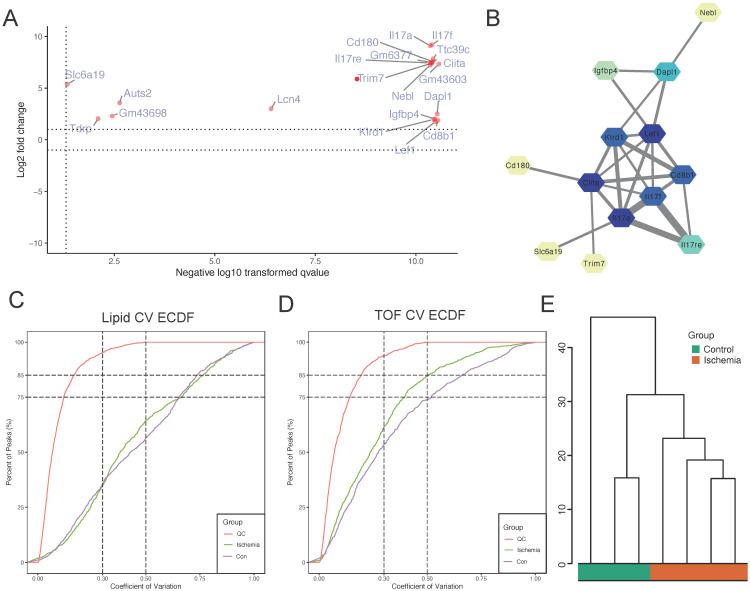
** Identification of core proteins and quality control of metabolomics sequencing data obtained on assessing samples from four patients with lower limb ischemia and three healthy controls.** (A) Scatter plot of differential expression of proteins in the “GOBP: positive regulation of phosphorus metabolic process” pathway. (B) Protein-protein interaction (PPI) network of proteins in this pathway. (C) Coefficient of variation of all lipids and (D) metabolites. (E) Hierarchical clustering analysis.

To investigate the proteins involved in “GOBP: positive regulation of phosphorus metabolic process,” we plotted a scatter plot with the differential expression of proteins involved in this pathway (Figure 4A). The expression levels of all proteins involved in this pathway were found to be upregulated. We also performed PPI analysis and found close interactions among Nebl, Dapl1, Igfbp4, Lef1, Klrd1, Ciita, Il17f, Cd8b1, Il17a, Cd180, Il17re, Trim7, and Slc6a19 (Figure 4B). These proteins seem to play a key role in influencing lower limb ischemia. To further explore the mechanisms of metabolism in lower limb ischemia development, we performed metabolomics sequencing (four patients with lower limb ischemia and three healthy controls). We performed quality control, and the CV plot indicated that the experimental data were very stable (Figure 4C-D). Hierarchical clustering analysis revealed significant differences between ischemic and normal samples (Figure 4E).

### Metabolic disorders and changes in lower limb ischemia progression

**Figure 5:**
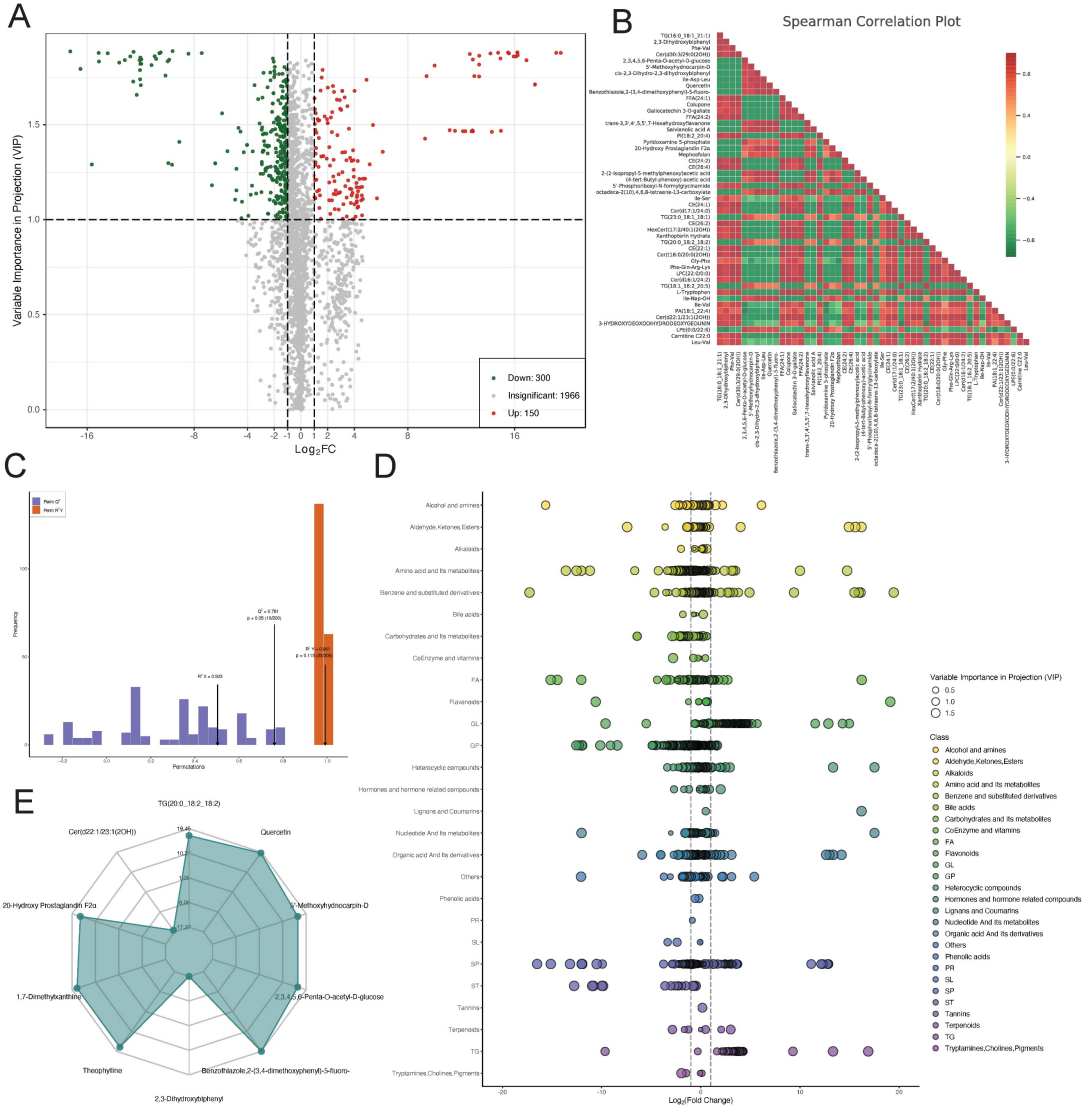
** Differential metabolite analysis.** (A) Volcano plot of differential metabolites. (B) Heatmap of correlations between differential metabolites. (C) OPLS-DA model validation plot. (D) Scatter and (E) radar plots of differential metabolites.

To explore the role of metabolites in the progression of lower limb ischemia, sequenced metabolites were subjected to differential analysis. A volcano plot was generated to visualize 300 downregulated metabolites, 150 upregulated metabolites, and 1,966 metabolites with relatively minor differences (Figure 5A). We then performed Spearman correlation analysis on the top 50 differentially expressed metabolites with the highest degree of difference. The results showed that there were more negative than positive correlations among these metabolites (Figure 5B). We validated the sequenced metabolites using an OPLS-DA model. The model exhibited an R2X of 0.503, R2Y of 0.991 (p = 0.115), and Q2 of 0.761 (p = 0.05), indicative of its effectiveness (Figure 5C). Scatter plots displayed the content of differential metabolites within different categories, showing significant differences in amino acid and its metabolites, suggesting that amino acid metabolism plays a key role in lower limb ischemia. Radar plots for the top 10 metabolites with the largest differential multiples provided further detail on these differences (Figure 5D-E).

### Association between key metabolites and clinical phenotypes of lower limb ischemia based on MR analysis

**Figure 6:**
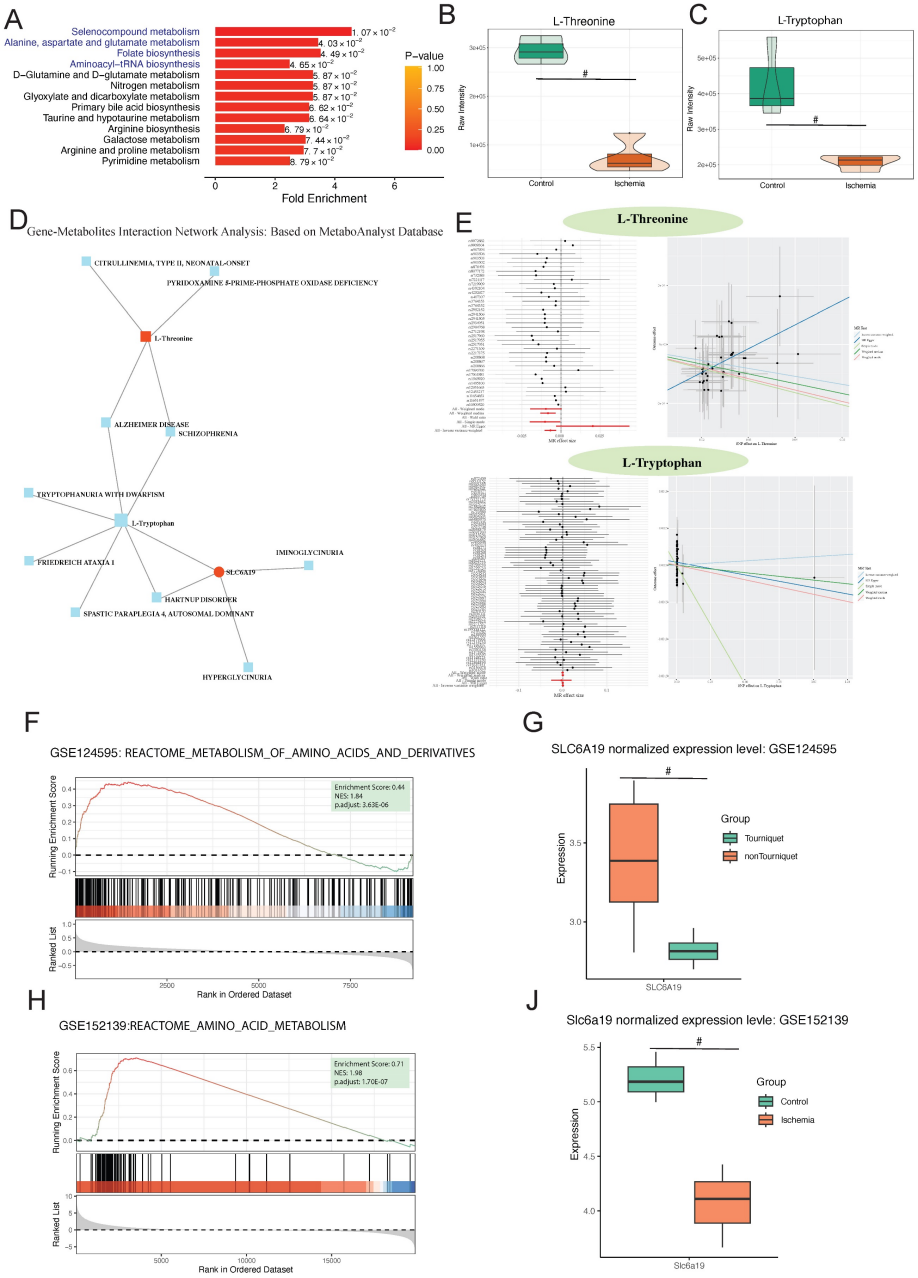
** Differential metabolite enrichment analysis, association between differential metabolites and genes, validation, and Mendelian randomization of metabolites.** (A) Metabolite set of differential metabolites. (B) Volcano plot of differential expression of L-threonine and (C) L-tryptophan. (D) Protein-metabolite interaction network. (E) Forest and scatter plots of Mendelian randomization results for L-threonine and L-tryptophan. (F) GSEA enrichment pathways in GSE124595. (G) Differential expression of SLC6A19 in GSE124595. (H) GSEA enrichment pathways in GSE152139. (I) Differential expression of SLC6A19 in GSE152139.

To reduce the omission of metabolites with important biological significance but not significant differential expression based on conventional enrichment analysis using the hypergeometric distribution, we performed metabolite set enrichment analysis to identify significantly different metabolite sets. Selenocompound metabolism was identified to be the most significantly enriched pathway (Figure 6A). Further analysis of the differentially expressed metabolites involved in this pathway identified L-threonine and L-tryptophan as showing the most significant differences (Figure 6B). We explored the interaction between these metabolites and hub genes, finding a close relationship with SLC6A19 (Figure 6D).

MR analysis with L-threonine and L-tryptophan as exposures and lower limb ischemia as outcomes showed that an increase in L-threonine reduces the risk of lower limb ischemia, indicating that L-threonine is a favorable factor for lower limb ischemia (IVW p = 0.003, MR-Egger p = 0.099, weighted median p = 0.009, simple model p = 0.057, and weighted model p = 0) (Figure 6E). To validate our findings, we conducted a thorough analysis of two transcriptomic datasets, GSE124595 and GSE152139. Our results revealed a significant enrichment of amino acid metabolism-related pathways in both datasets. Specifically, in GSE124595, the REACTOME_METABOLISM_OF_AMINO_ACIDS_AND_DERIVATIVES pathway was significantly enriched with an Enrichment Score (ES) of 0.44, a Normalized Enrichment Score (NES) of 1.84, and an adjusted p-value of 3.63E-06. Similarly, in GSE152139, the REACTOME_AMINO_ACID_METABOLISM pathway exhibited an ES of 0.71, an NES of 1.98, and an adjusted p-value of 1.70E-07 (Figure 6F; Figure 6H).

In addition, after normalization, the SLC6A19 gene was found to be significantly downregulated in the ischemic group across both datasets, with p-values of less than 0.05 in each case (Figure 6G; Figure 6J). This consistent downregulation further supports the involvement of amino acid metabolism in the observed ischemic responses.

## Discussion

In this study, we identified 13 key proteins using single-cell data from mice: Nebl, Dapl1, Igfbp4, Lef1, Klrd1, Ciita, Il17f, Cd8b1, Il17a, Cd180, Il17re, Trim7, and Slc6a19. On sequencing and analyzing samples from four patients with lower limb ischemia and three healthy controls, we identified L-threonine and L-tryptophan as key metabolites, along with their associated metabolic pathways. A close relationship was identified between SLC6A19 and L-threonine and L-tryptophan. We thus believe that SLC6A19 plays a key role in lower limb ischemia occurrence and development.

Succinic acid, a metabolite of the TCA cycle 41, plays a vital role in thrombus formation. It influences thrombus formation through multiple signaling pathways, including platelet aggregation, activation of coagulation factors, and endothelial dysfunction 42. According to the literature, succinic acid activates platelets by binding to succinate receptor 1 (SUCNR1), also known as GPR91. Activated SUCNR1 initiates endogenous signaling pathways, leading to the release of calcium, phosphorylation of kinases, and release of platelet activation factors, promoting platelet activation and aggregation, and ultimately leading to thrombus formation 43. Moreover, succinic acid participates in thrombus formation by regulating the activity of the coagulation system; it activates the coagulation cascade by promoting tissue factor expression on endothelial cells 44. Succinic acid-mediated tissue factor expression activates various transcription factors, such as nuclear factor κB, facilitating core enzyme cascades in blood clot formation 45. Some studies have also indicated that succinic acid regulates the expression of plasminogen activator inhibitor type 1, which plays a key role in balancing plasminogen and plasmin 46. Succinic acid further promotes thrombus formation by inducing the expression of P-selectin, a platelet-generated oligomer 47. Succinic acid also plays a crucial role in muscle injury repair caused by lower limb ischemia. It participates in protein synthesis and cell proliferation processes in muscle cell differentiation, aiding the synthesis of muscle fiber proteins and promoting muscle cell development and growth 48.

Tryptophan, an essential amino acid, plays a pivotal role in diverse metabolic processes. Tryptophan metabolism is intricately related to thrombus formation and development. Tryptophan produces key neurotransmitters, such as dopamine and serotonin, through its metabolic pathways, which play chief roles in thrombus formation 49, 50. Serotonin, by activating 5-hydroxytryptamine receptors on platelets, increases platelet aggregation and thrombus formation 51, while dopamine, via mechanisms such as increasing cAMP levels, reducing calcium levels in platelets, and inhibiting platelet activation, reduces platelet aggregation and activation, thereby inhibiting thrombus formation 52. Tryptophan metabolism is also closely related to the nitric oxide system. Nitric oxide is a potent vasodilator and antithrombotic agent that plays a significant regulatory role in thrombus formation 53. Tryptophan hydroxylase is a key enzyme regulating the rate of tryptophan metabolism, converting tryptophan into tyrosine while simultaneously releasing nitric oxide 54. Alterations in the tryptophan metabolic pathway may decrease the synthesis of nitric oxide, resulting in vascular contraction and platelet aggregation, eventually promoting thrombus formation 55. Tryptophan also plays a key role in muscle injury caused by lower limb ischemia. Tyrosine causes muscle damage by increasing reactive oxygen species levels, reducing muscle fiber size, inducing structural damage, and promoting lipid peroxidation, leading to muscle atrophy and functional decline 56. Tryptophan affects thrombus development by regulating the formation of serotonin, dopamine, and nitric oxide, playing a vital role in lower limb ischemia.

SLC6A19 closely interacts with tryptophan. B0AT1, encoded by *SLC6A19*, is a major transporter for free tryptophan 7. Therefore, SLC6A19 seems to participate in lower limb ischemia development, possibly by influencing tryptophan transport through differential expression, thereby affecting thrombus formation.

## Conclusion

To summarize, succinic acid and tryptophan play key roles in lower limb ischemia. A close interaction exists between them, with B0AT1, encoded by *SLC6A19*, potentially playing a crucial role by regulating tryptophan transport. Our analysis of single-cell data and metabolomics data revealed the significant roles of key genes and metabolites in the development of lower limb ischemia, enhancing our understanding of the pathogenesis of this condition and providing new insights into its prevention and treatment.

## References

[1] Angelini A, Cerchiaro M, Maturi C, Ruggieri P (2022). Vascular Complications Caused by Tibial Osteochondroma: Focus on the Literature and Presentation of a Popliteal Artery Thrombosis with Acute Lower Limb Ischemia. Diagnostics (Basel).

[2] Hess CN, Huang Z, Patel MR, Baumgartner I, Berger JS, Blomster JI (2019). Acute Limb Ischemia in Peripheral Artery Disease. Circulation.

[3] Natarajan B, Patel P, Mukherjee A (2020). Acute Lower Limb Ischemia-Etiology, Pathology, and Management. Int J Angiol.

[4] Chen L, Tian Q, Shi Z, Qiu Y, Lu Q, Liu C (2021). Melatonin Alleviates Cardiac Function in Sepsis-Caused Myocarditis via Maintenance of Mitochondrial Function. Front Nutr.

[5] Apichartpiyakul P, Shinlapawittayatorn K, Rerkasem K, Chattipakorn SC, Chattipakorn N (2022). Mechanisms and Interventions on Acute Lower Limb Ischemia/Reperfusion Injury: A Review and Insights from Cell to Clinical Investigations. Ann Vasc Surg.

[6] Willhite SR, Warner AC, Hain JS, Nickerson MC, Peterson DA, Cuddy DS (2022). Endovascular approach to thrombosed limb of aortoiliac endoprosthetic stent graft following abdominal endovascular aneurysm repair. J Vasc Surg Cases Innov Tech.

[7] Cuny H, Bozon K, Kirk RB, Sheng DZ, Bröer S, Dunwoodie SL (2023). Maternal heterozygosity of Slc6a19 causes metabolic perturbation and congenital NAD deficiency disorder in mice. Dis Model Mech.

[8] Stark K, Massberg S (2021). Interplay between inflammation and thrombosis in cardiovascular pathology. Nat Rev Cardiol.

[9] Chen L, Zhan CZ, Wang T, You H, Yao R (2020). Curcumin Inhibits the Proliferation, Migration, Invasion, and Apoptosis of Diffuse Large B-Cell Lymphoma Cell Line by Regulating MiR-21/VHL Axis. Yonsei Med J.

[10] Li X, Sim MMS, Wood JP (2020). Recent Insights Into the Regulation of Coagulation and Thrombosis. Arterioscler Thromb Vasc Biol.

[11] Huang Z, Yu P, Tang J (2020). Characterization of Triple-Negative Breast Cancer MDA-MB-231 Cell Spheroid Model. Onco Targets Ther.

[12] Grover SP, Mackman N (2019). Intrinsic Pathway of Coagulation and Thrombosis. Arterioscler Thromb Vasc Biol.

[13] Gao WL, Li XH, Dun XP, Jing XK, Yang K, Li YK (2020). Grape Seed Proanthocyanidin Extract Ameliorates Streptozotocin-induced Cognitive and Synaptic Plasticity Deficits by Inhibiting Oxidative Stress and Preserving AKT and ERK Activities. Curr Med Sci.

[14] Sun J, Singh P, Shami A, Kluza E, Pan M, Djordjevic D (2023). Spatial Transcriptional Mapping Reveals Site-Specific Pathways Underlying Human Atherosclerotic Plaque Rupture. J Am Coll Cardiol.

[15] Jiang L, Chen T, Xiong L, Xu JH, Gong AY, Dai B (2020). Knockdown of m6A methyltransferase METTL3 in gastric cancer cells results in suppression of cell proliferation. Oncol Lett.

[16] Udaya R, Sivakanesan R (2022). Synopsis of Biomarkers of Atheromatous Plaque Formation, Rupture and Thrombosis in the Diagnosis of Acute Coronary Syndromes. Curr Cardiol Rev.

[17] Peng Y, Wang Y, Zhou C, Mei W, Zeng C (2022). PI3K/Akt/mTOR Pathway and Its Role in Cancer Therapeutics: Are We Making Headway?. Front Oncol.

[18] Nishibe T, Dardik A, Kusakabe T, Fukuda S, Nishibe M, Koizumi J (2022). Association of lower limb ischemia with loss of skeletal muscle mass in patients with peripheral artery disease. Surg Today.

[19] Zeng J, Liu J, Ni H, Zhang L, Wang J, Li Y (2023). Mitochondrial transplantation reduces lower limb ischemia-reperfusion injury by increasing skeletal muscle energy and adipocyte browning. Mol Ther Methods Clin Dev.

[20] Ryan TE, Schmidt CA, Tarpey MD, Amorese AJ, Yamaguchi DJ, Goldberg EJ (2020). PFKFB3-mediated glycolysis rescues myopathic outcomes in the ischemic limb. JCI insight.

[21] Nemet I, Saha PP, Gupta N, Zhu W, Romano KA, Skye SM (2020). A Cardiovascular Disease-Linked Gut Microbial Metabolite Acts via Adrenergic Receptors. Cell.

[22] Shao Y, Zhao T, Zhang W, He J, Lu F, Cai Y (2020). Presence of the apolipoprotein E-ε4 allele is associated with an increased risk of sepsis progression. Sci Rep.

[23] Witkowski M, Weeks TL, Hazen SL (2020). Gut Microbiota and Cardiovascular Disease. Circ Res.

[24] Wen L, Cao Y, Cheng Q, Li X, Pan L, Li L (2020). Objectively measured near work, outdoor exposure and myopia in children. Br J Ophthalmol.

[25] Chen X, Wang M, Yu K, Xu S, Qiu P, Lyu Z (2023). Chronic stress-induced immune dysregulation in breast cancer: Implications of psychosocial factors. J Transl Int Med.

[26] Zhang L, Jiang B, Zhu N, Tao M, Jun Y, Chen X (2019). Mitotic checkpoint kinase Mps1/TTK predicts prognosis of colon cancer patients and regulates tumor proliferation and differentiation via PKCα/ERK1/2 and PI3K/Akt pathway. Med Oncol.

[27] Deng Y, Wang H, Guo X, Jiang S, Cai J (2023). Long-term blood pressure outcomes of laparoscopic adrenalectomy in trHTN patients. J Transl Int Med.

[28] Dou L, Lu E, Tian D, Li F, Deng L, Zhang Y (2023). Adrenomedullin induces cisplatin chemoresistance in ovarian cancer through reprogramming of glucose metabolism. J Transl Int Med.

[29] Kanehisa M, Furumichi M, Sato Y, Kawashima M, Ishiguro-Watanabe M (2023). KEGG for taxonomy-based analysis of pathways and genomes. Nucleic Acids Res.

[30] Aleksander SA, Balhoff J, Carbon S, Cherry JM, Drabkin HJ, Ebert D (2023). The Gene Ontology knowledgebase in 2023. Genetics.

[31] E IR, Bakarozi M, Dimas I, Galanis K, Lygoura V, N KG (2023). Total and individual PBC-40 scores are reliable for the assessment of health-related quality of life in Greek patients with primary biliary cholangitis. J Transl Int Med.

[32] Li J, Ma X, Lin H, Zhao S, Li B, Huang Y (2024). MHIF-MSEA: a novel model of miRNA set enrichment analysis based on multi-source heterogeneous information fusion. Front Genet.

[33] Reimand J, Isserlin R, Voisin V, Kucera M, Tannus-Lopes C, Rostamianfar A (2019). Pathway enrichment analysis and visualization of omics data using g:Profiler, GSEA, Cytoscape and EnrichmentMap. Nat Protoc.

[34] Liu Y, Liu Y, Ye S, Feng H, Ma L (2023). A new ferroptosis-related signature model including messenger RNAs and long non-coding RNAs predicts the prognosis of gastric cancer patients. J Transl Int Med.

[35] Luan Y, Huang E, Huang J, Yang Z, Zhou Z, Liu Y (2023). Serum myoglobin modulates kidney injury via inducing ferroptosis after exertional heatstroke. J Transl Int Med.

[36] Wang X, Chen JD (2023). Therapeutic potential and mechanisms of sacral nerve stimulation for gastrointestinal diseases. J Transl Int Med.

[37] Birney E (2022). Mendelian Randomization. Cold Spring Harb Perspect Med.

[38] Xu Z, Mo X, Kong Y, Wen Q, Han T, Lyu M (2023). Mini-dose methotrexate combined with methylprednisolone as a first-line treatment for acute graft-versus-host disease: A phase 2 trial. J Transl Int Med.

[39] Zhao Z, Jiao Y, Yang S, Zhou A, Zhao G, Guo S (2023). Endoscopic diagnosis and treatment of superficial non-ampullary duodenal epithelial tumors: A review. J Transl Int Med.

[40] Zhou B, Wang Z, Dou Q, Li W, Li Y, Yan Z (2023). Long-term outcomes of esophageal and gastric cancer patients with cardiovascular and metabolic diseases: A two-center propensity score-matched cohort study. J Transl Int Med.

[41] Jie XL, Luo ZR, Yu J, Tong ZR, Li QQ, Wu JH (2023). Pi-Pa-Run-Fei-Tang alleviates lung injury by modulating IL-6/JAK2/STAT3/IL-17 and PI3K/AKT/NF-κB signaling pathway and balancing Th17 and Treg in murine model of OVA-induced asthma. J Ethnopharmacol.

[42] Nock S, Karim E, Unsworth AJ (2023). Pim Kinases: Important Regulators of Cardiovascular Disease. Int J Mol Sci.

[43] Gilissen J, Jouret F, Pirotte B, Hanson J (2016). Insight into SUCNR1 (GPR91) structure and function. Pharmacol Ther.

[44] Zhang R, Lu S, Yang X, Li M, Jia H, Liao J (2021). miR-19a-3p downregulates tissue factor and functions as a potential therapeutic target for sepsis-induced disseminated intravascular coagulation. Biochem Pharmacol.

[45] Ekhlak M, Kulkarni PP, Singh V, Chaurasia SN, Mohapatra SK, Chaurasia RN (2023). Necroptosis executioner MLKL plays pivotal roles in agonist-induced platelet prothrombotic responses and lytic cell death in a temporal order. Cell death and differentiation.

[46] Morange PE, Peiretti F, Gourhant L, Proust C, Soukarieh O, Pulcrano-Nicolas AS (2021). A rare coding mutation in the MAST2 gene causes venous thrombosis in a French family with unexplained thrombophilia: The Breizh MAST2 Arg89Gln variant. PLoS Genet.

[47] Yousuf B, Pasha R, Pineault N, Ramirez-Arcos S (2022). Contamination of platelet concentrates with Staphylococcus aureus induces significant modulations in platelet functionality. Vox Sang.

[48] Wen ML, Wu P, Jiang WD, Liu Y, Wu CM, Zhong CB (2023). Dietary threonine improves muscle nutritional value and muscle hardness associated with collagen synthesis in grass carp (Ctenopharyngodon idella). Food Chem.

[49] Scott SA, Fu J, Chang PV (2024). Dopamine receptor D2 confers colonization resistance via microbial metabolites. Nature.

[50] De Giovanni M, Tam H, Valet C, Xu Y, Looney MR, Cyster JG (2022). GPR35 promotes neutrophil recruitment in response to serotonin metabolite 5-HIAA. Cell.

[51] Mammadova-Bach E, Mauler M, Braun A, Duerschmied D (2018). Autocrine and paracrine regulatory functions of platelet serotonin. Platelets.

[52] Joshi H, McIntyre WB, Kooner S, Rathbone M, Gabriele S, Gabriele J (2020). Decreased Expression of Cerebral Dopamine Neurotrophic Factor in Platelets of Stroke Patients. J Stroke Cerebrovasc Dis.

[53] Corro R, Urquijo CF, Aguila O, Villa E, Santana J, Rios A (2022). Use of Nitric Oxide Donor-Loaded Microbubble Destruction by Ultrasound in Thrombus Treatment. Molecules.

[54] Graboski AL, Kowalewski ME, Simpson JB, Cao X, Ha M, Zhang J (2023). Mechanism-based inhibition of gut microbial tryptophanases reduces serum indoxyl sulfate. Cell Chem Biol.

[55] Franczyk B, Gluba-Brzózka A, Ławiński J, Rysz-Górzyńska M, Rysz J (2021). Metabolomic Profile in Venous Thromboembolism (VTE). Metabolites.

[56] Kaiser H, Yu K, Pandya C, Mendhe B, Isales CM, McGee-Lawrence ME (2019). Kynurenine, a Tryptophan Metabolite That Increases with Age, Induces Muscle Atrophy and Lipid Peroxidation. Oxid Med Cell Longev.

